# Development of a motivational regulatory strategy scale for Indonesian learners of Japanese

**DOI:** 10.3389/fpsyg.2023.1245500

**Published:** 2023-09-29

**Authors:** Junko Yamashita

**Affiliations:** Seikei Institute for International Studies, Seikei University, Tokyo, Japan

**Keywords:** motivational regulatory strategies (MRS), Indonesian learners of Japanese, scale validation, integrative motivation, opportunity control

## Abstract

Opportunities for LOTE (Languages Other Than English) speakers to engage with their target language are limited, making it challenging to sustain motivation. The aim of this study is to develop and validate a scale for measuring motivational regulatory strategies among Japanese-language learners and investigate their relationship with motivational factors. This research specifically focuses on Indonesian learners of Japanese who have a non-Kanji (Chinese characters) background and are studying Japanese as a foreign language. The motivational regulatory strategies scale comprises six factors and has demonstrated adequate internal consistency and factor structure. The findings indicate a positive correlation between integrative motivation and these six strategies, suggesting that learners’ integrative motivation may promote the adoption of these strategies. Furthermore, this study emphasizes the significance of the opportunity control strategy, where learners actively seek chances to expose themselves to their target language. Future research is recommended to implement the developed scale in educational settings. Conducting surveys that encompass learners from diverse cultural backgrounds and embarking on longitudinal studies should also be considered.

## 1. Introduction

Learning English is becoming increasingly important in a globalized society. Meanwhile, over 3 million people worldwide are learning Japanese as a foreign language (Japan Foundation, 2020). In addition, the number of foreign nationals residing in Japan as of 2022 reached 2.96 million (Immigration Services Agency of Japan, 2022). Thus, there is an increasing demand for Japanese-language education both inside and outside Japan.

Japanese, as a LOTE, is not as extensively used on a global scale as English. Therefore, Japanese as a Foreign Language (JFL) learners often face difficulties in maintaining their motivation to learn Japanese, particularly in environments with limited opportunities for interaction with native Japanese speakers. Additionally, the intricate task of learning Kanji, with its multitude of character forms, pronunciations, and meanings, poses a significant challenge for those whose native writing systems are alphabetic, placing a heavy burden on students and demands substantial effort for mastery (Kano, 2017). As a result, the number of students in intermediate and advanced JFL classes at universities has significantly decreased compared to beginner-level classes (Yang, 2011; Northwood and Thomson, 2012). The largest number of Japanese learners with a non-Kanji background is in Indonesia. A decline in their motivation to learn Japanese has been reported in several studies conducted for Indonesian university students (e.g., Munakata and Quan, 2014; Yamashita, 2020a). Declining student’s motivation can cause them to abandon learning Japanese and change their future careers (Fatmawati, 2016). Hence, it is crucial to elucidate how these students should be able to sustain their motivation language learning that requires long-term continuity and effort management.

Motivational Regulatory Strategies (MRS) play an important role in managing and controlling one’s motivation (Dörnyei, 2001; Wolters, 2003). MRS refers to learners’ purposeful actions to influence their motivation intensity or the underlying processes for the pursuit of goals (Wolters, 2003). In addition, motivational variables that promotes the usage of MRS is another influential component (Wolters, 2003). The interrelationship between these two factors has received considerable attention (Cohen and Griffiths, 2015); however, the overarching relationship remains unclear (Teng et al., 2019). In Second Language Acquisition (SLA) field, MRS and motivational studies have only focused on English as a *lingua franca*, and research targeting Japanese language learners is limited. The language learning targeting TOLEs, such as Japanese, must be clarified to conceptualize motivation (Lanvers, 2016; Dörynei and Al-Hoorie, 2017).

Therefore, this study aims to develop a MRS scale for Japanese language learners to remain motivated and pursue learning. Additionally, this study sought to elucidate the relationship between MRS and motivation. This will contribute not only to the development of motivation and MRS research in Japanese language education but also to SLA research in terms of LOTE education.

## 2. Literature review

### 2.1. Self-regulated learning and MRS

Self-regulated learning refers to the process in which learners are actively involved in their own learning process in three aspects: cognitive, affective, and behavioral, to achieve their goals (Zimmerman and Schunk, 2008). Numerous empirical studies have proved that SRL has provided a solution to the challenge of how learners, as individual agents, participate in their own learning and effectively achieve their learning objectives. Learners with knowledge about learning strategies are more actively engaged in the learning process by setting goals, implementing learning strategies, monitoring, environment construction, and maintaining self-efficacy, which lead to enhanced academic achievement (Pintrich and De Groot, 1990; Zimmerman and Martinez-Pons, 1990). In the context of actively controlling the learning process, motivational regulation is recognized as one of the keys for a successful SRL (Boekaerts, 1995; Pintrich, 1999; Zimmerman and Schunk, 2008). MRS is necessary at each stage of learning - initiation, maintenance, and reflection upon completion of the task (Wolters, 2003), and it provides learners overcome difficulties and how to increase and maintain motivation (Zimmerman and Schunk, 2011). SRL includes a variety of strategies including cognitive and metacognitive strategies related to elaboration and critical thinking, but motivational regulation plays a distinct role in terms of regulation motivation.

Many researchers in the field of educational psychology have examined the critical importance of MRS as well as the scale development (e.g., Kuhl, 1985; Zimmerman and Martinez-Pons, 1986; Pintrich and De Groot, 1990; Corno and Kanfer, 1993; Pintrich et al., 1993; Wolters, 1998, 1999; McCann and Garcia, 1999; Ito and Shinto, 2003; Schwinger et al., 2009; Wolters and Benzon, 2013; Habók and Magyar, 2018). For example, in his early MRS study in 1998, Wolters categorized motivation-focused strategies within SRL and conducted research with 115 university students in the United States, examining how they maintained motivation in four situations: attending lectures, reading textbooks, writing papers, and studying for exams. The participants’ responses were categorized into four types of MRS: internal regulation (mastery goals, value, interest, and efficacy), external regulation (performance goals and extrinsic rewards), information processing (cognition and help seeking), and volition (environment, attention, willpower, and emotion). Additionally, he assessed their use of cognitive strategies (rehearsal, organization, elaboration, critical thinking, and metacognition) through a five-point questionnaire. Regression analysis was performed to examine the relationship between the four MRS and cognitive strategies. The results revealed that learners who maintained motivation through internal regulation employed more cognitive strategies such as elaboration and critical thinking. Interestingly, learners who used external regulation did not show significant tendencies toward any of cognitive strategies, yet they achieved higher grades compared to the internal regulation group. In later research (Wolters, 1999), Wolters utilized factor analysis to identify five MRSs (interest enhancement, performance self-talk, self-consequating, mastery self-talk, and environmental control). The outcomes of regression analysis disclosed that the combined MRSs accounted for approximately 16% of the variance in students’ semester GPA, with only performance self-talk being identified as a significant predictor. Wolters’ findings highlighted that differences in intrinsic and extrinsic motivational regulation strategies can lead to varying effects on the use of cognitive strategies and academic performance. Building on Wolters’ study, Schwinger et al. (2009) further elaborated on MRS through a self-report survey conducted with 231 German high school students and identified the following eight distinct strategies: enhancement of situational interest, enhancement of personal significance, mastery self-talk, performance-approach self-talk, performance-avoidance self-talk, self-conquering, proximal goal setting, and environment control. The results of pass analysis revealed a positive correlation between the use of MRS and effort management, subsequently associated with GPA. Nevertheless, it was found that does not directly lead to better academic performance. Besides, McCann and Garcia (1999) categorized the MRS scale based on Kuhl’s (1985) concept of volition. They developed Academic Volitional Strategies Inventory (AVSI) questionnaire, which focused on the maintenance of motivation and the regulation of emotions within the academic learning environment of a university setting. AVSI consists of self-efficacy enhancement (e.g., believing in one’s ability to understand and remember course material, the positive outcomes of completing assignments), stress reduction (e.g., promise oneself a reward for completing a specific amount of study, playing background music to create a relaxing atmosphere), and negative-based incentives (e.g., thinking about the disappointment of others if one performs poorly, considering the amount of time classmates spend studying).

Previous studies have found similar scales across these context despite variations in participants’ environment whether high school and college. For instance, Wolters (1998) interest enhancement is categorized similarly in Schwinger et al. (2009), though it was further divided into situational and personal aspects. Moreover, both research shares the same concept of self-talk based on mastery or performance. Self-consequating corresponds to the exact same self-rewarding scale, so does environment control. A distinct strategy introduced by Schwinger et al. (2009) is proximal goal setting. Furthermore, when contrasting with McCann and Garcia (1999), their three broader categories encompass multiple strategies. For instance, self-efficacy enhancement includes enhancement of personal significance and mastery self-talk. Similarly, stress reduction encompasses both self-consequating and environment control. Negative-based incentives is the sole exception, aligning exclusively with performance self-talk in Schwinger et al. (2009).

Considering the assessment of MRS, previous studies have not consistently found a positive and direct correlation between MRS and academic achievement. This is because the concept of MRS encompasses knowledge and skills aimed at maintaining and enhancing learners’ motivation (Dörnyei, 2001; Wolters, 2003). While strategies directly influencing cognitive activities might have a direct impact on grades or GPA, the primary focus of employing MRS is to enhance motivation by fostering increased effort and sustained learning (Schwinger et al., 2009). Rather, learners’ motivational characteristics play a crucial role for deeper understanding of MRS, considering that motivation and MRS are distinct but interrelated (Wolters, 2003). Therefore, this study addresses motivation as a factor related to MRS usage. Similar studies examining the relationship between motivation and MRS are also observed in SLA, and these will be discussed in detail in the following section.

### 2.2. MRS in second language learning

Dörnyei (2001) was the pioneering researcher to have brought MRS to the SLA field. Similar to McCann and Garcia (1999), he applied Kuhl’s (1985) volitional theory and categorized five strategies: commitment control, metacognitive control, satiation control, emotional control, and environment control. Commitment control is aimed at sustaining learning for goal achievement (e.g., keeping promising expectations in mind, considering what to do when goals are not met). Metacognitive control involves learners objectively monitoring their own learning, controlling learning to avoid procrastination (e.g., eliminating distractions for better study habits, focusing on the initial stages of learning). Satiation control reduces boredom during learning and strive to make learning enjoyable (e.g., adding fun to activities, using methods that make learning enjoyable). Emotion control involves seeking a change in mood during learning to regain concentration (e.g., self-encouragement, engaging in relaxation techniques). Environmental control indicates making use one’s surroundings to sustain the learning process (e.g., eliminating obstacles to learning, arranging the environment for better concentration). Learners employ these five categories to boost their motivation when facing a loss of interest or when learning does not proceed as planned. Later, Tseng et al. (2006) constructed item scales based on Dörnyei’s classification, exampled as “I have special techniques to achieve my learning goals.” The reason for adopting such an expression is that SLA strategy research has typically revealed correlations between strategies and performance, indicating the usefulness of these strategies. Yamamori et al. (2003) criticized that questionnaire items asking about the extent to which the strategies were used as they can be interpreted as implying that greater use of strategies would result in more successful language acquisition. In response to these criticisms, Tseng et al. constructed items asking whether learners possess the ability to use the strategy, rather than its frequency. They argued that this would allow for the interpretation that learners who are aware of strategy skills are more likely to succeed in language acquisition, as opposed to learners who frequently use successful strategies for language acquisition.

Dörnyei’s (2001) taxonomy was also applied in the context of Japanese-language learning by Rose and Harbon’s (2013) work. They conducted interviews throughout a year with Australian learners of Japanese in order to gain a full picture of motivational regulation in Kanji learning. The researchers followed the methodology by Tseng et al. (2006) and illustrate how learners use MRS to sustain motivation by formulating the network codes based on Dörnyei’s (2001) taxonomy: commitment control (short-term goals, long-term goals), metacognitive control (procrastination, time management), satiation control (boredom, interest), emotional control (stress, self-criticism, defeatism, frustrations), environmental controls (time, place, people). The results indicated that learners with higher proficiency face more challenges in achieving tasks related to kanji learning compared to those with lower proficiency, highlighting the greater importance of employing MRS to address these difficulties. They emphasized the importance of setting short-term goals, effective time management, and gaining opportunities to encounter kanji, such as utilizing authentic reading materials in kanji learning through their mention that “kanji learning is a lifelong task (p. 103).”

One notable point is the definitional fuzziness of environmental control. Based on interview data, they pointed out that environmental control could be a strategy used to achieve other MRS, such as creating a distraction-free environment to reduce procrastination. One reason for this is that Tseng et al.’s items use expressions like “I know how to arrange the environment.” It becomes difficult for researchers to determine whether this refers to studying in a preferred location, at a specific time, or for a specific reason to change the environment. Since this expression does not sufficiently specify learners’ actions, details about “how to arrange the environment” could be interpreted differently. The ambiguity of the term “environment” and the lack of an explicit purpose for changing the environment highlight the difficulty in treating environmental control as a standalone category. In contrast, the concept of environmental control developed in educational psychology literature (e.g., Wolters, 1999) provides a clearer presentation of the content and strategies of environmental control, as exemplified by statements like “I change my surroundings so that it is easy to concentrate on the work.” Overly abstract scale items may cause problems when actually providing learning support (Ito and Shinto, 2003). Therefore, expressions in MRS items should follow the lead of educational psychology research and provide clearer indications of their content.

### 2.3. Link between MRS and motivation in SLA

In the realm of SLA, similar to educational psychology, similar to educational psychology, many researchers are attempting to capture the effects of self-regulation ability on the acquisition of foreign languages. The mainstream of research predominantly employs quantitative methods to develop SRL strategies and examine their impact on academic performance and language proficiency enhancement. For instance, Ardasheva (2016) conducted a questionnaire survey with primary and secondary English language learners residing in the United States to investigate the relationship between SRL strategies and English proficiency. The results of structural equation modeling revealed that metacognitive strategy had a direct impact on English proficiency and demonstrated the facilitative effect of SRL strategies on language learning ability. Teng and Zhang (2016) developed a questionnaire scale called Writing Strategies for Self-Regulated Learning Questionnaire (WSSRLQ) to measure learners’ SRL strategies in L2 writing context. They additionally examined the how these strategies influence their writing performance. The nine writing strategies explained 37% of the variance in participants’ writing test results, further contributing to providing evidence of the relationship between SRL usage and language learning achievement.

Regarding of MRS, previous studies have investigated the relationship between learners’ motivational factors and the characteristics of MRS to clarify how they regulate language learning. This differs from educational psychology literature, where MRS itself is categorized based on the nature of strategies, such as intrinsic vs. extrinsic (Wolters, 1998), mastery vs. performance (Wolters, 1999; Schwinger et al., 2009), and performance-approach vs. performance-avoidance (Schwinger et al., 2009). SLA has traditionally advanced motivation research through a sociocultural approach by Gardner and Lambert’s (1959, 1972) work: integrative and instrumental motivation. Integrative motivation refers to language learners’ desire for integrating into a culture or society through learning the target language. It emphasizes the goal of participating in different cultures or communities and communicating with others through language. On the other hand, instrumental motivation refers to the motivation that language learners pursue specific goals or benefits by learning the language. Learning a language for work, successful achievement on a text and exam, or for business or job-related purposes falls under this type of motivation. In the case of English learners, driven by the current worldwide demands of English language skills, they no longer desire to integrate into a specific culture or society. Instead, international posture driven by motivations for intercultural communication, such as international employment and interaction with people from different cultures, is being recognized as a major motivational component (Yashima, 2000). Kormos and Csizér (2014) conducted a study to investigate how motivation among Hungarian English learners contributes to their effective utilization of MRS, enabling them to engage in successful autonomous learning outside the classroom. Employing structural equation modeling, they introduced a model that explains the connection between motivational factors, self-regulating strategies, and autonomous learning behaviors. The motivational elements encompassed international posture and instrumental motivation. As for MRS, they selected three factors based on Mezei’s (2008) qualitative study conducted in the Hungarian context: satiation control from Dörnyei’s (2001) taxonomy, time management (controlling procrastination and plan their study schedule: falls under the category of metacognitive control), and opportunity control (seeking out opportunities for language exposure, such as watching English TV and reading magazines, and prioritizing English in daily communication). Concerning other MRS from Tseng et al. (2006), they excluded environmental control and emotional control due to insufficient reliability in their pilot study. The resulting model from their study provided a mixed outcome: among university students and adult learners, the self-concept shaped by international posture and instrumental motivation influenced SRL strategies. Conversely, in the case of high school students, only international posture directly correlated with self-concept. They concluded that strong international and instrumental motivation shapes learners’ self-concept as successful language users, thereby facilitating autonomous learning through the mediation of MRS. They particularly highlighted the significance of a proactive attitude toward finding learning opportunities as a crucial factor for autonomous learning.

Overall, the research has revealed how having (and what type of) motivation is crucial, and how such motivation can be maintained through the utilization of MRS. It highlights the importance of investigating the relationship between motivation and MRS, given the varying impacts of language learners’ learning environments and motivation toward the target language. However, similar to the discussion presented by Rose and Harbon (2013), there remains a question about Tseng et al. (2006) MRS items, as observed in the study by Kormos and Csizér (2014). Due to the unclear content of the items in Tseng et al. (2006) scale, it lacks sufficient reliability for use in research, leading to ambiguity in its definition as well. Ensuring both validity and reliability is essential for the robust development of a questionnaire Ensuring the validity and reliability of a questionnaire is essential for its robust development and suitability for future research like strategy instruction.

### 2.4. Motivation among Japanese language learners and its relationship with MRS

As highlighted in the preceding section, motivation plays an essential role in language learning, shaping the learning process, fostering active learner engagement, and promoting persistence. Consequently, the study of motivation gained significant attention, particularly among JFL learners who demand a heightened need for motivation. Early research by Nuibe et al. (1995) adopted Gardner and Lambert’s items to measure integrative and instrumental motivation. This scale has been applied to students from various countries beyond the regions of Asia, Europe, and Oceania (Nuibe et al., 1995; Narita, 1998; Guo and Okita, 2001; Guo and Quan, 2006; Onishi, 2010; Yang, 2011; Nemoto, 2012). Common categories consistently emerged, including integrative and instrumental motivation derived from Gardner and Lambert’s work, pop-culture orientation (with learners intrigued by Japanese popular culture and aim to enhance their comprehension and appreciation), interpersonal orientation (enthusiastic engagement with Japanese individuals to foster cultural understanding and establish connections), and language learning interest (learners enthusiasm to improve communication skills through learning Japanese). Focusing on Indonesia, Japanese language learning is positioned as a crucial aspect of cultivating international talent in the global society, given its significance in various career paths such as employment in Japanese corporations, studying abroad, and roles as interpreters, translators, and Japanese language instructors (Furukawa et al., 2015). Influenced by pop culture, career opportunities that involve Japanese proficiency have become more diverse, leading to a heightened enthusiasm for learning Japanese among Indonesian learners. Building upon prior research conducted with JFL learners, Yamashita (2020b) devised a motivational scale suitable for measuring Indonesian learners’ motivation. The results of exploratory factor analysis and confirmatory factor analysis showed that the scale, comprising six factors, was successfully validated and demonstrated satisfactory reliability. The six dimensions were: cultural understanding orientation, interpersonal orientation, language-learning orientation, pop-culture orientation, career orientation, and grade orientation. A noticeable difference from earlier research is the splitting of instrumental motivation into two categories: career orientation and grade orientation. This division aligns with the findings of Guo and Okita (2001) and Guo and Quan (2006), where instrumental motivation was separated into career and academic grade aspects due to the strong motivation for high-grade achievement among Chinese-speaking learners. While quantitative research has attempted to categorize motivational orientations, qualitative studies have used interviews to explore which specific motivations within these categories contribute to Japanese language learning. For example, de Burgh-Hirabe (2019) interviewed university students in New Zealand who learn Japanese, revealing that integrative motivation strongly influence on their Japanese learning. Similarly, Nomura and Yuan (2019), focusing on Japanese language learners in Hong Kong, found that along with integrative motivation, interest in pop culture serves as a reason for choosing to learn Japanese. The international status of English has led to a decreasing integrative motivation toward specific cultures among English learners, whereas for learners in LOTEs contexts, including Japanese, the desire to connect with the culture of the target language continues to play an essential role (Kwok and Carson, 2018).

Although MRS research for Japanese language learners remains under development, Yang (2011) investigated the relationship between Taiwanese learners’ motivations and efficacy enhancement, one of MRS. This study found that career orientation, grade orientation, and exchange orientation promoted the use of efficacy enhancement. Similar to the findings with English learners (Kormos and Csizér, 2014; Zheng et al., 2018), Taiwanese learners also demonstrated a strong career orientation while employing MRS in their Japanese language learning process. In Yang’s study, however, neither integrative motivation (cultural understanding orientation, language-learning orientation) nor pop-culture orientation showed any enhancing effects on MRS. Despite prior research highlighted that integrative motivation and pop culture orientation have a significant impact on learning Japanese (de Burgh-Hirabe, 2019; Nomura and Yuan, 2019), these findings suggest that their influence may not necessarily extend to the realm of MRS application. Considering Yang’s focus on Taiwanese learners with a background in Chinese characters, there is also a possibility that instrumental motivation, as observed in works like Guo and Okita (2001) and Guo and Quan (2006), strongly affected the usage of MRS. This could be attributed to the lack of validity confirmation, since Yang only classified the MRS scale through exploratory factor analysis and calculated alpha coefficients without any further item analysis. In any case, research concerning MRS in the context of Japanese language learning is still its early stages, requiring further exploration for a deeper understanding. Therefore, more comprehensive research involving participants from diverse cultural backgrounds is essential, along with the creation of a theoretically grounded instrument to reconfirm the connection between MRS and motivation. This approach would help enhance our understanding of MRS’s role and effectiveness in the Japanese language learning process.

## 3. Methods

Research on MRS targeting Japanese language learners is limited, and there is a lack of understanding of how learners in a JFL environment can effectively maintain motivation. To assess their MRS acquirement and utilize it for providing strategy instruction, there is a need for a scale that can measure strategies. However, existing MRS scales targeting SLA learners have certain limitations, warranting the development of a new scale. Moreover, due to differences in the motivational characteristics of English learners and Japanese learners, it is crucial to investigate the relationship between MRS and motivation to uncover the role of MRS in Japanese language learning.

In Study 1 of this research, an exploratory factor analysis was conducted using survey data from 157 Indonesian Japanese language learners to examine the structure of MRS. Concurrently, Cronbach’s alpha and McDonald’s omega reliability coefficients were calculated to assess the internal consistency of the factor structure. In Study 2, a separate questionnaire survey was administered to 221 Indonesian Japanese language learners. Firstly, a confirmatory factor analysis was performed to validate the factorial structure, aligning with Messick’s (1995) structural aspects. Additionally, Pearson correlation coefficients were computed to assess the correlation between MRS and motivation. If integrative motivation proves to influence MRS among Japanese language learners, it could offer valuable insights into motivational research within the context of LOTE learners, an area that has received limited attention in SLA research.

### 3.1. Participants

The study involved 157 Indonesian learners who were enlisted from universities in Bandung and Malang, both located on the island of Java. The participants’ ages ranged from 18 to 26 (*M* = 20.16, *SD* = 1.157), with 39% males (*n* = 61) and 61% (*n* = 96). The participants were categorized based on their academic year as follows: 35 students were in their first year, 102 students were in their second year, 42 students were in their third year, and 29 students were in their fourth year. There were no participants with study experience in Japan; all participants were confirmed to be learners in a JFL environment. Participants’ proficiency levels were classified based on their results in the Japanese Language Proficiency Test (JLPT). The JLPT assesses language knowledge, listening, and reading, without including speaking and writing components. Participants’ proficiency levels are divided into five levels, ranging from the easiest N5 to the highest level N1. Since the test does not assess participants’ speaking and writing skills, making a direct comparison with the Common European Framework of Reference for Languages (CEFR) is difficult. However, generally, N5 and N4 are estimated to correspond to beginner levels (A1, A2), N3 to intermediate level B1, and N2 and N1 are equivalent to B2 level. In Study 1, participant distribution was as follows: No certification = 22, N5 passed = 21, N4 passed = 31, N3 passed = 54, N2 passed = 26, N1 passed = 3.

In Study 2, 221 university students from universities in Bandung and Malang on the island of Java participated, constituting a different group from those in Study 1. The participants’ ages ranged from 18 to 25 (*M* = 19.72, *SD* = 1.407), with 43% males (*n* = 94) and 57% (*n* = 127). The breakdown of participants by grade was as follows: 85 first-year students, 61 s-year students, 65 third-year students, and 10 fourth-year students. Again, there were no participants with study experience in Japan; all participants were confirmed to be learners in a JFL environment. In Study 2, participants’ Japanese Language Proficiency Test (JLPT) achievements were as follows: No certification = 78, N5 passed = 51, N4 passed = 38, N3 passed = 39, N2 passed = 15. The demographic characteristics of participants in each study are presented in Table 1.

**Table 1:** Demographic characteristics of participants.

	Study 1	Study 2
Number of participants	157	221
Age (range)	18–26 (*M* = 20.16 ± 1.16)	18–25 (*M* = 19.72 ± 1.41)
Gender		
Male	61 (39%)	94 (43%)
Female	96 (61%)	127 (57%)
Academic year		
1st year	35	85
2nd year	102	61
3rd year	42	65
4th year	29	10
Japanese Language Proficiency Test (JLPT)		
No certification	22	78
N5 passed	21	51
N4 passed	31	38
N3 passed	54	39
N2 passed	26	15
N1 passed	3	0

### 3.2. Instruments

#### 3.2.1. MRS

Dörnyei’s (2001) categories are grounded in prior research in educational psychology, categorizing MRS for second language learners. Originally, this represented an innovative application in the field of SLA. However, with the increased recognition of SRL among SLA researchers, new MRS factors like opportunity control have been identified through investigations with L2 learners (Mezei, 2008; Kormos and Csizér, 2014). Building upon the framework of previous studies, this research aims to conceptualize six MRS factors by incorporating opportunity control into Dörnyei’s categories. As mentioned in Sections 2.2 and 2.3, there is an issue with the ambiguity of items created by Tseng et al. (2006). Given that Dörnyei’s classification draws on prior psychological research, there exists a certain overlap between his five categories and the items related to psychological MRS. To address the issue of definitional fuzziness, this study attempts to refine the items and enhance the reliability of the scale by drawing on previous psychological literature.

The generation of questionnaire items proceeded as follows: For each of Dörnyei’s (2001) five categories, corresponding scales were identified, and their item expressions were referred to. Considering contextual differences, the terminology within the questions was modified to be suitable for Japanese language learning. Below are the prior studies corresponding to the five categories along with sample questions: Commitment control aims to sustain learning by expecting desired outcomes or imagining situations where goals are not achieved. It aligns with the concept of Self-talk in McCann and Garcia (1999), Wolters (1999), and Schwinger et al. (2009). Six items were created based on their work (e.g., I think about how great I’ll feel when I get this assignment finished). Metacognitive control involves learners objectively assess their learning progress and employ strategies to avoid procrastination. This aligns with time management in Kormos and Csizér (2014) and Zheng et al. (2018). Therefore, six items related to time management were created (e.g., I set short-and long-term goals for Japanese language study). Satiation control tackles the issue of boredom during learning and aims to make it enjoyable. A similar strategy is interest enhancement (Wolters, 1999), and thus, five items were constructed applying his scale (e.g., I devise ways to make studying Japanese more enjoyable). Emotion control involves seeking a change in mood during learning to regain concentration, this strategy includes self-encouragement. McCann and Garcia’s (1999) self-efficacy enhancement also aligns with this concept. Consequently, six items were created based on their work (e.g., I tell myself that I can understand what I learn in class). Environmental control involves learners modifying their study environment to promote continuous learning. The concepts of environmental control are well articulated in the works of Wolters (1999) and Schwinger et al. (2009) Thus, five items were created based on their studies (e.g., I take a break so that it is easy to concentrate on my Japanese study). Additionally, opportunity control was added to the above categories. Five items related to opportunity control were developed based on Mezei (2008) and Kormos and Csizér (2014) (e.g., I try to make time to use Japanese voluntarily). Following the guidance from previous research, a total of 33 MRS were created. To better suit Japanese as a Foreign Language (JFL) learners, specific items were modified as necessary. Responses were collected using a 7-point scale (1: Strongly disagree) to (7: Strongly agree).

#### 3.2.2. Motivation

This study targets university students in Indonesia who are learning Japanese in the JFL environment. Yamashita (2020b) has developed a motivation scale for Japanese language learning from the same participant pool, demonstrated the sufficient validity and reliability (Cronbach’s alpha coefficients = 0.75–0.86) of the six-factor scale through multiple factor analyses. Therefore, in Study 2, this motivational scale was utilized to examine the relationship with MRS. The scale comprises six factors: cultural understanding orientation (e.g., Studying Japanese helps me to understand Japanese culture), interpersonal orientation (e.g., To communicate with Japanese people in Japanese), language learning orientation (e.g., Because I am interested in language learning itself), career orientation (e.g., Because Japanese is advantageous for my future career), grade orientation (e.g., Because I need to study Japanese to achieve good grades), and pop-culture orientation (e.g., Because I am interested in Japanese pop culture). Participants were asked to rate the extent to which the items were consistent with their motivation in response to the question “Why are you studying Japanese?” using a 7-point scale ranging from 1 (not at all agree) to 7 (very much agree).

### 3.3. Procedures

Before conducting the survey, the study was reviewed and approved by the Ethical Review Committee of the author’s institution at the time. The first survey for Study 1 was conducted in September 2019. Participants were informed that their cooperation in the survey was voluntary, that they can withdraw from the survey at any time, and that their cooperation would not affect their university grades. Two advanced Japanese language learners of Indonesian (JLPT N1 obtained) were asked to perform back-translation to eliminate ambiguous expressions and discrepancies between the questionnaire items in both languages.

The second survey for Study 2 was conducted between February and March 2020. The flow of the survey was similar to that of Survey 1. The Indonesian version of the items was used in the survey and translated by the same two translators as in Study 1.

## 4. Results

### 4.1. Exploratory factor analysis of MRS

First, the mean, standard deviation, kurtosis, and skewness of the 33 items were calculated. Four items that indicated ceiling or floor effects were eliminated, and an exploratory factor analysis (R ver. 3.5.0) with maximum likelihood and a Promax rotation were performed on the remaining 29 items. The eigenvalues varied from 7.17, 2.07, 1.45, 1.36, 1.17, to 0.97; therefore, a six-factor structure was adopted because the eigenvalue of the sixth factor was approximately 1.00. Items with loadings below 0.35 for each factor and six items with loadings on multiple factors were eliminated. The final factor loadings are presented in Table 2.

**Table 2:** Factor loadings for MRS and Cronbach’s alpha and Mcdonald’s omega coefficients.

	1	2	3	4	5	6
Factor 1: Self-talk (*α* = 0.77, *ω* = 0.80)						
I imagine the sense of accomplishment after completing assignments.	0.75	−0.05	0.00	0.07	−0.03	−0.04
I think about how great I’ll feel when I get this assignment finished.	0.66	−0.09	0.14	0.09	−0.05	−0.02
I remind myself that I will get a good grade if I follow my study plan.	0.58	0.14	−0.01	0.13	0.02	0.21
I think that my classmates will study harder and get better grades than me.	0.55	0.12	−0.01	−0.27	−0.03	0.15
I think of times when I have procrastinated and failed on past exams.	0.49	0.03	−0.08	−0.01	0.15	−0.02
Factor 2: Time management (*α* = 0.80, *ω* = 0.81)						
I set short-and long-term goals for Japanese language study.	0.02	0.83	−0.03	0.05	−0.06	−0.09
I allocate time to study on my own outside of class.	−0.04	0.74	0.04	−0.06	0.13	−0.03
I establish a daily schedule so that I have time to study Japanese.	0.08	0.66	0.16	−0.02	0.05	−0.07
I set realistic deadlines for my goals and make sure I achieve them by then.	0.04	0.54	−0.11	0.07	−0.05	0.07
Factor 3: Opportunity control (*α* = 0.78, *ω* = 0.82)						
I try to make time to use Japanese voluntarily.	0.07	−0.09	0.87	−0.09	0.09	0.00
I actively use the grammar and vocabulary I learned in daily conversation.	0.02	0.15	0.66	−0.05	−0.09	−0.03
When I meet Japanese, I try to speak in Japanese as much as possible.	0.00	−0.11	0.58	0.18	−0.04	0.06
I try to voluntarily increase my exposure to the Japanese language.	−0.12	0.24	0.52	0.07	0.05	0.02
Factor 4: Efficacy enhancement (*α* = 0.73, *ω* = 0.75)						
I will tackle hard tasks with the confidence that I can do them.	−0.03	0.02	0.05	0.71	−0.06	−0.02
I gained more confidence that I can achieve the goals I have set for myself.	0.03	0.00	−0.07	0.65	0.07	−0.07
When I do not make any progress in my studies, I tell myself I can do it.	−0.17	0.16	−0.03	0.55	0.03	0.20
I tell myself that I can understand what I learn in class.	0.21	−0.07	0.15	0.47	0.02	−0.01
Factor 5: Environmental structuring (*α* = 0.71, *ω* = 0.74)						
I choose a place with few distractions for studying Japanese.	0.01	−0.02	0.00	0.02	0.98	−0.07
I change the place where I study to change my mood.	−0.02	0.00	0.14	−0.03	0.38	0.16
I take a break so that it is easy to concentrate on my Japanese study.	0.16	0.13	−0.15	0.04	0.37	0.20
Factor 6: Interest enhancement (*α* = 0.71, *ω* = 0.72)						
I think of a way to make studying more interesting.	0.11	−0.07	−0.08	−0.02	0.03	0.87
I make my Japanese learning enjoyable by incorporating fun elements.	−0.11	−0.11	0.25	0.00	0.10	0.54
I devise ways to make studying Japanese more enjoyable.	0.00	0.29	0.07	0.02	−0.16	0.46

Factor 1 was named “self-talk” to imagine when the task or study was completed. Factor 2 was named “time management” because it comprised items that ensured study time and deadlines to achieve the goal. Factor 3 was named “opportunity control” because it includes items that ensured opportunities to speak Japanese or to be exposed to it. Factor 4 was named “efficacy enhancement” because it indicates a strategy to increase motivation by gaining a sense of self efficacy. The fifth factor was named “environmental structuring,” because adjusted the studying environment for the better concentration. The sixth factor was named “interest enhancement” because it comprised items to devise study methods for making study enjoyable and fun.

### 4.2. Reliability of MRS

Cronbach’s alpha coefficients and McDonald’s omega coefficients for each subscale were calculated to examine the internal consistency of the scales. Sufficient internal consistency was confirmed for all scales: self-talk (*α* = 0.77, *ω* = 0.80), time management (*α* = 0.80, *ω* = 0.81), opportunity control (*α* = 0.78, *ω* = 0.82), efficacy enhancement (*α* = 0.73, *ω* = 0.75), environmental structuring (*α* = 0.71, *ω* = 0.74), and interest enhancement (*α* = 0.71, *ω* = 0.72). The two types of coefficients values are presented in Table 2.

### 4.3. Validity of MRS

**Figure 1:**
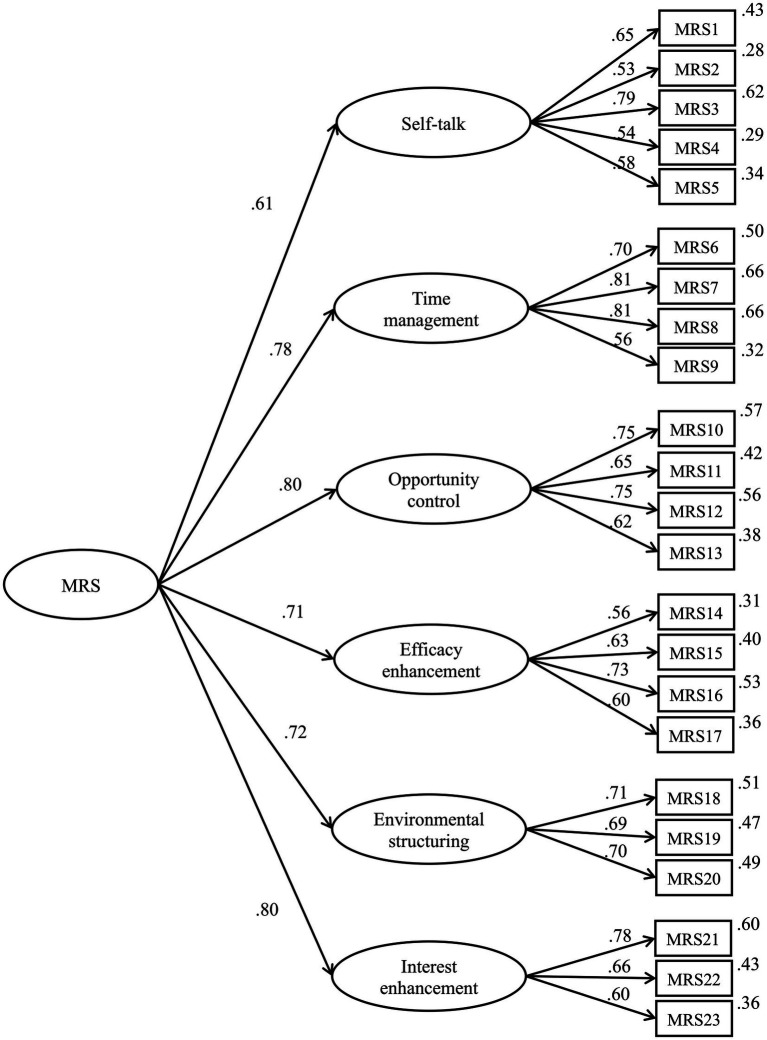
Result of confirmatory factor analysis.

A confirmatory factor analysis was conducted to validate the factor structure obtained from the exploratory factor analysis (see Figure 1). The indices obtained, including *χ*^2^*/df* = 1.805, CFI = 0.91, IFI = 0.91, and RMSEA = 0.06 [0.051, 0.070], were all within the acceptable range.

### 4.4. Factor correlation analysis

Prior to the correlation analysis, we computed the mean, standard deviation, and internal consistency (Cronbach’s alpha and McDonald’s omega coefficient) of MRS and motivation. These descriptive statistics are presented in Table 3.

**Table 3:** Descriptive statistics of MRS and the motivational scale.

	*M*	*SD*	α	ω
MRS				
Self-talk	5.48	1.08	0.78	0.79
Time management	4.48	1.11	0.82	0.84
Opportunity control	4.89	1.07	0.78	0.81
Efficacy enhancement	5.24	0.99	0.72	0.76
Environmental structuring	4.93	1.18	0.71	0.74
Interest enhancement	5.37	1.00	0.70	0.74
Motivation				
Cultural understanding orientation	5.52	1.14	0.70	0.71
Interpersonal orientation	5.20	1.28	0.81	0.82
Language learning orientation	5.86	1.01	0.80	0.80
Career orientation	5.74	1.05	0.83	0.84
Grade orientation	5.09	1.65	0.84	0.85
Pop-culture orientation	5.19	1.53	0.87	0.87

Pearson’s correlation coefficients were calculated for the MRS and the motivation scale (see Table 4). First, all MRS were positively correlated with integrative motivation, including cultural understanding orientation (*r* = 0.32–0.46) and interpersonal orientation (*r* = 0.29–0.49). A positive correlation was also found with language learning orientation (*r* = 0.29–0.49) and career orientation (*r = 0*.25–0.35). For grade orientation, a weak positive correlation was found with self-talk (*r* = 0.25), and positive correlations were found with time management (*r* = 0.12) and environmental structuring (*r* = 0.13) but not with the other three strategies. Pop-culture orientation was positively correlated with time management (*r = 0.*13), opportunity control (*r* = 0.42), efficacy enhancement (*r* = 0.21), and interest enhancement (*r* = 0.22) but not with self-talk and environmental structuring.

**Table 4:** Correlation coefficients between MRS and the motivational scale.

	Cultural understanding	Interpersonal	Language learning	Career	Grade	Pop-culture
Self-talk	0.32***	0.30***	0.46***	0.35***	0.25***	0.01
Time management	0.33***	0.33***	0.35***	0.27***	0.12*	0.13*
Opportunity control	0.46***	0.49***	0.49***	0.27***	−0.04	0.42***
Efficacy enhancement	0.37***	0.40***	0.45***	0.33***	0.06	0.21***
Environmental structuring	0.33***	0.29***	0.29***	0.25***	0.13**	0.10
Interest enhancement	0.39***	0.35***	0.35***	0.32***	0.02	0.22***

*p < 0.05, **p < 0.01, ***p < 0.001.

## 5. Discussion

### 5.1. MRS for Japanese language learning

The present study primarily aimed to develop and validate an MRS scale for assessing learners’ strategy usages in Japanese language learning. Previous MRS scales targeting language learners were based on Dörnyei’s (2001) categories, and the scale developed by Tseng et al. (2006) gained wide recognition. However, its items lacked precision in their expressions, resulting in concerns regarding reliability and definitional clarity. To address this issue, specific action-oriented expressions were incorporated into each item, drawing from MRS scales developed in educational psychology studies by Wolters (1999), Schwinger et al. (2009), and McCann and Garcia (1999). As a result, exploratory factor analyses confirmed the six-factor structure of the scale, which included self-talk, time management, opportunity control, efficacy enhancement, environmental structuring, and interest enhancement. Factor 1, self-talk, refers to how learners persuade themselves for goal achievement and to motivate themselves when facing difficulties or a lack of enthusiasm in Japanese learning. This factor aligns with similar strategies found in educational psychology (McCann and Garcia, 1999; Wolters, 1999; Schwinger et al., 2009), where a more detailed categorization has been made based on the content of self-talk in their research. This study revealed that both positive and negative self-talk items were included in the same factor. This suggests that the act of engaging in self-talk, whether the outcomes of learning or exams are positive or negative, functions as a mechanism for controlling motivation among Indonesian Japanese language learners. Factor 2, time management, involves learners objectively assessing their learning progress and employing strategies to avoid procrastination. Procrastination is a common challenge for individuals, but Indonesian university students use this time management strategy to lower psychological barriers to Japanese learning and mitigate demotivational. This strategy has been observed in other language learning contexts, such as Hungarian English learners (Kormos and Csizér, 2014) and Chinese English learners (Zheng et al., 2018), indicating its widespread applicability. Building on Mezei’s (2008) and Kormos and Csizér’s (2014) insights, this study incorporated opportunity control as the third factor strategy, considering its value for Indonesian learners of Japanese. JFL environment provides limited opportunities for exposure to Japanese and contact with Japanese speakers. Indonesian university students actively seek chances to use Japanese, recognizing the value and importance of learning the target language. The findings of this study have contributed to establishing the value of this strategy as a broadly applicable MRS in language learning. Factor 4, labeled as efficacy enhancement, refers to students enhancing their self-efficacy to maintain motivation for goal attainment. Higher levels of self-efficacy lead to enhanced performance abilities in SRL (Zimmerman and Schunk, 2011), and this phenomenon is also applicable to language learning (e.g., Mizumoto, 2011). The results of this study similarly indicate that Indonesian university students enhance their self-efficacy for motivational control, which may contribute to achieving their goals in Japanese language learning. Factor 5, environmental structuring, involves learners’ effort to create a better learning environment to enhances concentration, as observed in educational psychology (Wolters, 1999; Schwinger et al., 2009). The existence of this category indicates that Indonesian university students make efforts to sustain their concentration by changing locations or taking breaks, serving as a means to maintain or enhance their motivation. The final factor, interest enhancement, describes how students increase their interest in Japanese learning, aiming to make it more enjoyable. This strategy is primarily employed to reduce learners’ boredom, as observed in Dörnyei (2001) and Wolters (1999) work. In the context of long-term language learning, students often encounter challenging or less engaging tasks, which can lead to a demotivation. In such situations, Indonesian university students use this strategy to manage their motivation.

The MRS scale, which includes the six strategies mentioned above, underwent reliability assessment using two different reliability measures. In the case of questionnaire scales, alpha values exceeding 0.70 are considered favorable, as recommended by Takeuchi and Mizumoto (2014). The alpha reliability of each factor was determined to surpass this threshold, showing adequate internal consistency. Next, the indices obtained from confirmatory factor analyses fell within an acceptable range. Through two types of factor analyses, a valid and reliable scale specialized for motivational regulation in language learning has been developed in the field of SLA. The findings suggest that Indonesian university students studying Japanese in JFL context are likely to employ these six strategies when facing challenges or setbacks in their Japanese learning journey, thereby maintaining their motivation.

### 5.2. Relationship to motivation scale

In SLA, there has been a concerted effort to understand the characteristics of MRS by exploring their connection with learners’ motivational factors. Previous studies have focused on English language learners and emphasized the international role of English as a *lingua franca*, as demonstrated in Kormos and Csizér (2014). Therefore, as the second objective, the present study examined the relationship between Indonesian learners of Japanese and their motivational factors. The results reported that all MRS factors were positively correlated with integrative motivation (cultural understanding orientation and interpersonal orientation), and language learning orientation. The correlational values observed between these three motivational factors and MRS fall within the range of either moderate or weak correlation, yet they exhibit statistically significant positive connections with all six MRS strategies, particularly with opportunity control showing a relatively strong correlation. This finding provides empirical evidence for the relationship between integrative motivation and the use of MRS, suggesting that integrative motivation plays a crucial role in regulating motivation among Japanese language learners. This result aligns with previous research by de Burgh-Hirabe (2019) and Nomura and Yuan (2019), emphasizing the significance of integrative motivation for Japanese learners and other LOTEs. In addition, the findings in the study provide important insights for a better understanding of MRS in SLA contexts. Compared to other strategies, opportunity control was found to have stronger correlations with integrative motivation scales. This suggests that the strategy, which is not categorized in Dörnyei’s (2001) framework, is found to be an important strategy for language learners. While the theoretical application of educational psychology literacy is useful, we should not overlook the fact that there are strategies that can only be found in SLA environments where language learning is investigated. The present study shows that the opportunity control, which played a major role in regulating motivation through Mezei’s (2008) in-depth interviews and Kormos and Csizér’s (2014) large-scale study, can also be observed in learners beyond the target language. In conclusion, Indonesian learners’ attitude that maintain and improve their motivation to see, hear, and speak Japanese is closely related to positive attitudes toward target language speakers, Japanese people, and Japanese language speakers, Japanese people, and Japanese society.

Instrumental motivation yielded varying outcomes, depending on the construct. Firstly, career orientation was positively correlated with all strategies, which was consistent with previous studies by Kormos and Csizér (2014) and Yang (2011), highlighting a causal relationship between college students’ career orientation and the use of MRS. This study also focused on university students majoring in Japanese. The results further corroborate the relationship between the motivation to apply the learned language in future careers and the facilitation of SRL. Regarding grade orientation, the results showed a weak correlation with self-talk, time management, and environmental structuring, but not with the other three strategies. Notably, the correlation coefficients were lower compared to those associated with other motivational factors. This result suggests that SRL is unlikely to be facilitated by grade orientation in Indonesian learners of Japanese. Interestingly, these results contradict the findings of Yang’s (2011) study, which identified a relationship between grade orientation and efficacy enhancement. One possible explanation for this discrepancy is the selection of Taiwanese Japanese learners by Yang. For learners with a Kanji background, Japanese shares the same writing system as their native language, which could lead to a reduced learning burden compared to learners from alphabetic language backgrounds like English. Furthermore, in East Asian countries, including Taiwan, there is a strong emphasis on education and competition for admission to prestigious institutions, placing significant value on academic achievements (Sasaki, 2018). Such a competitive educational environment might have amplified the impact of instrumental motivation, as observed in studies like Guo and Okita (2001) and Guo and Quan (2006). Motivation is a complex individual factor that varies based on learning environments. Considering that it also influences the use of MRS, it becomes imperative to explore the characteristics of MRS by investigating the relationship between learners’ context, motivation, and their usage of MRS. This study, which focused on Indonesian learners with a non-Kanji background, highlights the strength of integrative motivation. In order to gain a thorough grasp of the intricate connection between motivation and MRS, it is anticipated that research will involve learners from various cultural backgrounds.

Finally, pop-culture orientation were positively correlated with four MRSs (time management, opportunity control, efficacy enhancement, and interest enhancement) except for self-talk and environmental structuring. While it was highly correlated with opportunity control, the coefficient values with other three MRSs were relatively low, indicating a similar tendency to grade orientation. According to Nomura and Yuan (2019), Hong Kong students study Japanese language due to integrative motivation and an interest in pop culture. However, its influence does not extend to the regulation of motivation throughout the learning process. In other words, the influence of interest in pop culture on MRS is limited, unlike integrative and career orientation. The results of the high correlation between pop culture orientation and opportunity control indicate that interest in pop culture affects motivational regulation in a different dimension from the specific learning process in SRL. Given that opportunities to come across Japanese in Indonesian islands such as Bandung and Malang are limited, learners’ interest in Japanese pop culture may have triggered them to incorporate Japanese into their daily lives, which may have increased the correlation coefficient between the two. Conversely, no correlation was found between self-talk, which has keywords “assignments” and “exam,” or between environmental structuring. Given that pop culture, such as anime and manga, is a form of entertainment for Japanese learners (Nemoto, 2012), it is assumed that they do not utilize pop culture to engage in Japanese learning, assignments, or concentration in their studies.

In summary, this study’s findings strengthen the connection between motivation and MRS and emphasize motivation as a significant factor influencing motivational regulation in the SRL process. Additionally, these results highlight the crucial role of integrative motivation in enhancing motivational regulation among Indonesian learners of Japanese, particularly for LOTE language learners. Nevertheless, the impact of grade orientation appeared relatively small and distinct from that observed in Taiwanese learners of Japanese. Future research involving participants from diverse native language and cultural backgrounds is warranted to provide a more comprehensive understanding of motivation and MRS. Regarding pop-culture orientation, our study revealed that it plays an essential role for generating interest in Japanese. However, it may not necessarily promote the use of all MRSs. Given that different types of motivation can influence MRS in various ways, it’s crucial to closely examine the entire SRL process, including how motivation is managed, and conduct numerous studies over time to progressively unravel this intricate relationship.

## 6. Conclusion

When learning a LOTE, it can be challenging to recognize the value of the language and pursue career prospects. In regions where opportunities to engage with the target language are limited, there is a greater need to actively seek opportunities for language interaction. In Indonesia, the decrease in motivation to learn Japanese among university students has been pointed out by Munakata and Quan (2014) and Yamashita (2020a). Therefore, there was an urgent need to identify ways to prevent and maintain students’ motivation in long-term language learning. The developed questionnaire scale in this study has been demonstrated to encompass crucial constructs for measuring the use of MRS in the context of Japanese language learning. The primary significance of this research lies in providing empirical evidence that integrative motivation remains a vital factor related to the utilization of MRS for LOTE learners. It also highlights the crucial role of actively seeking opportunities to engage with the target language for these learners in maintaining their motivation. One key advantage of our research is the application of the questionnaire, as it can be implemented in various aspects of students’ learning processes within classroom environments. For instance, it enables teachers to assess students’ progress in skill acquisition, providing insights into classroom trends. Additionally, it serves as a self-assessment tool, affording learners the opportunity to understand how to maintain their motivation and reflect on their learning. Furthermore, it can be employed to instruct strategies as scaffolding for learners who have limited experience with MRS.

### 6.1. Limitations and implications for future research

This study has two main limitations. The first limitation concerns participant selection. While this study focused on Indonesian university students majoring in Japanese, there is a diverse group of individuals learning Japanese, including high school students, adults, and those with a heritage language background. Furthermore, among Japanese learners in a JFL environment, there is diversity in learners’ first language, and educational policies also vary from country to country. Despite this study has emphasized the significance of integrative motivation, the relationship between motivation and MRS produced different results compared to prior research. To better understand the underlying reasons, future research should explore ways to uncover the link between motivation and MRS, encompassing learners with different language background and in various classroom environments.

The second limitation is that this study did not determine whether learners were actually able to execute SRL through the MRS employment. To address this issue, it is necessary to explore the relationship between MRS and other SRL factors, including cognitive aspects that have a direct impact on their performance. Moreover, for a comprehensive conceptualization, relying solely on a one-time questionnaire survey may be insufficient. To truly grasp the impact of MRS, it is crucial to conduct classroom investigations and longitudinal studies. These future research not only contributes to clarify the conceptualization of SRL in Japanese learning but also provides valuable insights from the perspective of LOTE education, which can be beneficial for SLA studies.

## Data availability statement

The raw data supporting the conclusions of this article will be made available by the authors, without undue reservation.

## Ethics statement

The studies involving humans were approved by the Hiroshima University Ethics Review Committee. The studies were conducted in accordance with the local legislation and institutional requirements. The participants provided their written informed consent to participate in this study.

## Author contributions

The author confirms being the sole contributor of this work and has approved it for publication.

## Funding

Preparation of this article was facilitated by a research grant (Grant-in-Aid for Early-Career Scientists 22K13153) to the author from the Japan Society for the Promotion of Science.

## Conflict of interest

The author declares that the research was conducted in the absence of any commercial or financial relationships that could be construed as a potential conflict of interest.

## Publisher’s note

All claims expressed in this article are solely those of the authors and do not necessarily represent those of their affiliated organizations, or those of the publisher, the editors and the reviewers. Any product that may be evaluated in this article, or claim that may be made by its manufacturer, is not guaranteed or endorsed by the publisher.
